# Health mediation does not reduce the readmission rate of frequent users of emergency departments living in precarious conditions: what lessons can be learned from this randomised controlled trial?

**DOI:** 10.1186/s12873-024-01000-2

**Published:** 2024-05-15

**Authors:** Michel Rotily, Nicolas Persico, Aurore Lamouroux, Ana Cristina Rojas-Vergara, Anderson Loundou, Mohamed Boucekine, Themistoklis Apostolidis, Sophie Odena, Celia Chischportich, Pascal Auquier

**Affiliations:** 1 Centre d’Etudes et de Recherche sur les services de santé et la qualité de vie (CEReSS), Aix Marseille Université, Marseille, France; 2 Service des Urgences, Hôpital Nord, Assistance Publique Hôpitaux de Marseille, Marseille, France; 3 Centre de santé hospitalo-universitaire des Aygalades, Assistance Publique Hôpitaux de Marseille, Marseille, France; 4 Laboratoire de Psychologie Sociale (LPS), Aix Marseille Université, Aix en Provence, Marseille, France; 5 Laboratoire d’Economie et de Sociologie du Travail, Aix Marseille Université, Centre National de la Recherche Scientifique, Aix en Provence, Marseille, France; 6 SCOP Regards Santé, Marseille, France

## Abstract

**Background:**

Severe overcrowding of emergency departments (EDs) affects the quality of healthcare. One factor of overcrowding is precariousness, but it has rarely been considered a key factor in designing interventions to improve ED care. Health mediation (HM) aims to facilitate access to rights, prevention, and care for the most vulnerable persons and to raise awareness among healthcare providers about obstacles in accessing healthcare. The primary aim was to determine whether HM intervention for frequent users of EDs (FUED) living in precarious conditions could reduce the readmission rate at 90 days.

**Methods:**

Between February 2019 and May 2022, we enrolled and interviewed 726 FUED in four EDs of southeastern France in this randomised controlled trial. The HM intervention started in the ED and lasted 90 days. In addition to the primary endpoint (first readmission at 90 days), secondary endpoints (readmission at 30 and 180 days, number of hospitalisations at 30, 90, 180 days, admissions for the same reasons as the first admission) were also studied. The outcomes were measured in the ED information systems. Statistical methods included an intention-to-treat analysis and a per-protocol analysis. Comparisons were adjusted for gender, age, ED, and health mediator.

**Results:**

46% of patients reported attending the ED because they felt their life was in danger, and 42% had been referred to the ED by the emergency medical dispatch centre or their GP; 40% of patients were considered to be in a serious condition by ED physicians. The proportion of patients who were readmitted at 90 days was high but did not differ between the control and the HM intervention groups (31.7% vs. 36.3%, *p* = 0.23). There was no significant difference in any of the secondary outcome measures between the control and HM intervention groups. Per-protocol analysis also showed no significant difference for the primary and secondary endpoints.

**Conclusions:**

This randomised controlled trial did not show that our health mediation intervention was effective in reducing the use of emergency services by FUED living in precarious conditions. Some limitations are discussed: the duration of the intervention (90 days), the long-term effects (> 6 months), the involvement of the ED staff.

**Trial registration:**

Registered on clinicaltrials.gov as NCT03660215 on 4th September 2018.

## Background

Iterative use of Emergency Departments (EDs) is a major topic in health services research. This affects the quality and efficiency of care, results in lost opportunities for patients, and creates financial losses [1–4]. Many researchers have attempted to identify the characteristics of FUED. These characteristics are many and varied, even between studies and study populations: age, social isolation, chronic diseases and comorbidities, psychiatric problems, economic hardship, being unemployed or dependent on government welfare, being uninsured, living closer to ED [5–10]. Many of these characteristics affect people living in precarious conditions. Social interventions, personalised and coordinated care, and health education to improve health empowerment could be of benefit to this population as well as to EDs [11]. However, interventional studies in these populations are lacking and the overall effectiveness of strategies to reduce the readmission rate of FUED is still under debate [12–14].

### Health mediation to reduce health inequities

Health mediation (HM) is one of the key strategies that the French government has put in place to combat health inequities [15]. HM emerged at the end of the 80s for patients living with AIDS and mental diseases [16]. HM is intended to be a proximity interface aimed, on the one hand at providing access to rights, to prevention and to care for populations presenting various factors of vulnerability that distance them from the health systems, and on the other hand at raising awareness in the actors of the health system to the specificities of these populations and to the obstacles they encounter in their healthcare pathways [16, 17]. HM is based on the major principles of “going towards” populations, health and social professionals and institutions and “doing with” in a logic of empowerment of individuals [16, 18]. In some countries, work has been done with professionals close to health mediators (HMrs), such as community health workers (CHW) highlighting the benefits of their interventions in hospitals [19, 20]. In some ways, HMrs are close to CHW, in the sense that they serve as a link between health/social services and the community to facilitate access to services and improve quality-of-service delivery. HMrs also build individual and community capacity by increasing health knowledge and self-sufficiency through a range of activities such as outreach, education, informal counselling and social support. However, HMrs are not always trusted members of the community served or the neighbourhoods, and they do not provide education, support or advocacy at a community level but only at the individual level. Although HM has been widely promoted by the French Ministry of Health and many actors in the healthcare system, and its implementation has been evaluated in the context of health promotion, of the management of chronic and mental illnesses and of the access to health in vulnerable people, tangible data are not available on its effectiveness on access to health, and the quality and efficiency of health care in these populations [16, 21–24].

An HM intervention targeting deprived FUED, starting in ED and consisting of education actions and help on navigating the care system could reduce readmissions to ED. After the examinations, care and recommendations have been made, patients living in precarious conditions are generally discharged from EDs with a report to their general practitioner (which they do not always have), one or more prescriptions for tests or medication that they do not always understand or that they do not know how to carry out, cannot or do not want to carry out, or do not identify as priorities. Emergency physicians and most nurses do not have specific training or the time to determine psychosocial needs and most do not know what resources are out in the community to fill in the gaps. Social services are attached to EDs but are not sufficiently staffed and trained in empowerment, care pathways, health literacy and outreach techniques [25]. Although HM seems appropriate to reduce iterative ED use in this population, HM has never been evaluated in this context.

### A need for tools adapted to deprived frequent users of emergency departments

The two main strategies tested to reduce iterative ED use are accompaniment by CHWs and, in particular, case management. Very few studies have reported on the impact of CHW on FUED. Yet CHW could help leverage EDs as an entry point into the healthcare system, reduce costs per patients, improve overall health outcomes and elevate some of the ED physicians’ responsibility [26–28]. Several systematic reviews suggested that case management could reduce ED visits [12, 14, 29, 30] and be cost-effective [31], but few specifically targeted vulnerable patients [13, 29, 30]. Case management involves multi-disciplinary teams including physicians, nurses, psychologists, social workers and/or housing and community resource liaisons, who develop tailored care strategies and protocols for patients [32–35]. Little is known about the impact of case management on deprived FUED, and interventional trials in this population are crucial.

To address the issue of iterative use of EDs among persons living in precarious conditions, we set up a research project whose main objective was to evaluate the effectiveness of HM in EDs on the readmission rate of this population. We have already shown that HM is well accepted by patients and ED staff alike, but its efficacy remains to be proved [36]. The primary aim of this study was to demonstrate that health mediation intervention for FUED living in precarious conditions can reduce the readmission rate in EDs at 90 days. The secondary objectives were to evaluate the impact of HM on the readmission rates in EDs at 30 and 180 days, and on the number of readmissions in EDs and hospitalisations at 30, 90 and 180 days.

## Methods

### Study population

This two-arm parallel randomised controlled trial was conducted in four EDs in southeastern France. Two EDs were in densely populated urban areas with high levels of precariousness (North University Hospital, and European Hospital, in northern and central districts of Marseille); the two other EDs were in less urbanized areas characterised by pockets of neo-rural precariousness (Arles and Manosque). Each year, these 4 EDs handle 23% of all ED stays in the counties where they are located; 1,830,495 people aged over 18 live in these counties, with a density of 183 inhabitants per km² and a poverty rate of 17.8% (such as defined by the National Institute for Statistics and Economic studies). The annual use of EDs is 42% (number of ED stays/population aged 18 and over). These four EDs provide mainly medical and surgical health care (95%, including 29% for traumas); psychiatry and toxicology represents 4% of ED stays [37].

Patient enrolment began in February 2019, and the last patient was enrolled in November 2021. The last follow-up was in May 2022. Men and women presenting to one of the participating EDs were eligible to participate if they were at least 18 years old; had visited the same ED once in the 90 days prior to enrolment, or twice in the last 6 months, or three or more times in the last 12 months; were able to communicate in French (even if they could only speak French poorly); understood the purpose of the study; and had an EPICES social precariousness score greater than 30. The EPICES score estimates the level of precariousness using 11 binary items: marital status (one item), health insurance status (one item), economic status (three items), family support (three items) and leisure activity. It can vary from 0 (no precariousness) to 100 (extreme precariousness), with 30 being the cut-off point for classifying people as being in a precarious situation [38]. .

Patients were not included if they were unable to give informed consent, could not respond to a face-to-face interview (confused, acutely psychotic, with severe neurodegenerative disorders or intoxicated), were under guardianship, legal protection or imprisonment, were living in a nursing home or other health and social care facility with a care team, or were in a life-threatening emergency situation. Patients living in an area initially considered too remote for the mediator to visit were also excluded.

The health mediators (HMrs) (see below) assigned to the study screened all patients attending the ED (including those who had attended the previous evening or during the night and were still present in the ED) for age, place of residence, and whether they had attended the ED during the period used as an inclusion criterion. HMrs were present on weekdays between 7 am and 8 pm, excluding Saturdays, Sundays and public holidays. Each patient with no exclusion criteria was interviewed to complete the EPICES social precariousness questionnaire. If the EPICES score was compatible with the inclusion criterion and the medical staff did not object on medical grounds, the patient was given full information about the study and the intervention procedures, and signed a written consent form.

### Collected information

All patients were interviewed by HMrs to collect socio-demographic characteristics: age, gender, residence, distance to ED, marital status, education level, occupational status, income and migration, public and complementary health insurance coverage, allocation of various allowances, access to general practitioner, quality of life (WHOQOL-Bref) and reason for ED admission. Initial complaints, severity score, main and related pathologies, discharge mode (return home or hospitalisation) and duration of hospitalisation were collected from the hospital information system at baseline, 30, 90 and 180 days. The initial complaints were classified using the thesaurus of the French Society of Emergency Medicine (SFMU), and the final diagnoses were matched according to their International Classification of Diseases (ICD-10) codes. We used the SFMU severity score to classify patients into 5 categories (1–2: lesion status and/or functional prognosis judged stable, without (1) or with (2) further diagnostic or therapeutic action required; 3: lesion status and/or functional prognosis judged likely to deteriorate in the ED, but not life-threatening; 4–5: life-threatening pathological situation, without (4) or with (5) resuscitation techniques). The REDCAP software was used to enter all the information collected [39].

### Health mediation intervention

Enrolled patients were randomised to one of two arms at the time of their ED visit: ‘control’ (usual care) or ‘experimental’ (HM), by the HMrs according to a randomisation list generated by an independent statistician using a 4-block randomisation prior to the start of the study, for each of the four EDs, and implemented in the REDCAP database software [40]. Once a patient met the study inclusion criteria, was informed of the study objectives and procedures, and signed the informed consent, the HMr entered data from the EPICES social precariousness questionnaire into the REDCAP software, which checked this score (30+) and told the HMr which group (control or experimental) the patient had been allocated to. Randomisation therefore took place immediately after enrolment on computers in the EDs of the participating hospitals. In the experimental arm, the patient was managed by an HMr from the time of admission to the ED and followed up for 90 days, in accordance with the recommendations of the French High Authority for Health [18]; examples of HM are displayed in Table 1.

**Table 1:** Examples of health mediation

Case 1: Mrs X., 74 years old, came to the ED several times in the last six months for the same reason, a high blood pressure. She reported regular meetings with the GP and the cardiologist, with a nurse visiting every day to check her blood pressure and medications. Mrs X. and her husband were both on full social security coverage.
The couple live on a small pension. At the time of the initial interview by the HM, Mrs X. stated that she had been having financial problems for 3 months due to the suspension of housing allowances. The husband had been to the family benefits fund office several times, but had not been able to resolve the problem. The loss of support meant that the couple’s resources were significantly reduced, making it difficult for them to pay the rent or buy food. This situation was a great source of stress for Mrs X.
The HM put the couple in touch with a social worker to look for a solution regarding housing benefits and to find social housing. After several weeks, the couple recovered their housing benefit. In the meantime, the HM informed Mrs X. about the various food distribution associations near her home and called her regularly to check on her and reassure her. Mrs X. did not return to the ED in the following months.
CASE 2: Mr X., aged 58, regularly returns to the ED for several reasons (chronic bronchitis, depressive syndrome, alcohol abuse). He is very isolated, out of touch with the health care system, has neither a regular general practitioner, nor complementary medical insurance, nor exemption from fees for a long-term illness. Mr X. does not receive housing benefits because he has no rental agreement and pays low rent in an unhealthy dwelling in danger of collapsing. Mr X. has a very low income, receiving financial allowance for his disability. He has a large debt with the hospital and refuses any contact with a social worker.
The mediation lasted 90 days. Several meetings took place at home and at the hospital, with dozens of telephone calls. The patient was reintegrated into a care programme with a GP in his neighbourhood, obtained recognition of his chronic pathologies for full healthcare insurance, and the hospital’s litigation department was informed in order to regularise his debts. He obtained help with the payment of supplementary health insurance and was able to start dental and ophthalmic treatment. Finally, he was evacuated from his home and rehoused, and a social follow-up with an association was launched. A neighbourhood citizens’ association keeps in touch with Mr X. to break his isolation. The HM had to call on several structures outside the hospital to find the best alternatives for his complex situation. Mr X. reduced the number of ED admissions from 15 in the 6 months prior to inclusion to 4 in the 90 days following inclusion.

HMr: Health Mediator; ED: Emergency Department

The five HMrs were full-time paid staff with 2–5 years post-baccalaureate education and a diploma in social work, with some experience in the health sector; medical background was not required. Qualified applicants were selected on the basis of good communication skills, good knowledge of social rights and procedures and common health care pathways, ability/experience in teamwork and networking with health/social professionals inside and outside the EDs, and in managing relationships with disadvantaged people in an ethical and equitable manner. They were initially trained and supervised by a senior HMr (for attitudes and behaviour towards these people) and a general practitioner, with routine group or individual meetings to present challenging cases.

The tasks of HMrs consisted of (1) administering a questionnaire on socio-demographics, quality of life, health literacy, and reasons of admission to ED; (2) evaluating the socio-medical needs of patients according to ad hoc guidelines; (3) defining objectives corresponding to activities and resources of the services requested; (4) accompanying persons towards prevention and care, and helping them understand how to access social and health care; (5) acting as an interpreter and bridge to the persons concerned but also to health professionals and social workers; (6) adopting a benevolent stance and active listening in order to detect individual and collective problems that might require specific information or prevention. All of these tasks were carried out with a view to improving the capacity for health empowerment of the patients.

### Measurements – outcomes

Primary outcome: first readmission in ED at 90 days after inclusion.

Secondary outcomes: ED readmission at 30 and 180 days, number of ED readmissions at 30, 90 and 180 days, number of hospitalisations after ED readmission at 90 days, admission to ED at 30, 90 and 180 days for the same reason as the first admission.

### Sample size

The sample size was calculated to detect an efficacy of the intervention on the primary endpoint: 4% in the intervention group versus 10% in the control group, with an attrition rate of 15%, a significance level of 0.05 and power of 0.80. Based on these assumptions, the total required sample size was estimated to be 726 patients (363 subjects per group).

### Statistical methods

Groups were compared from their initial allocation, regardless of adherence to the HM intervention (intention-to-treat analysis); a second analysis was performed (per-protocol analysis) comparing the primary and secondary endpoints in the control group to those in patients who met the HMrs at least once face-to-face after inclusion, at least three times by phone, and did not abandon the intervention during the 90-day follow up by the HM. A subgroup analysis in per-protocol patients was performed on patients with low severity scores (CCMU 1–2), and then on patients living in very precarious conditions (EPICES score 60+). Continuous variables are expressed as means and SDs or as median with range (min-max), and categorical variables are reported as count and percentages. Comparisons of mean values between two groups were performed using student t-test or Mann-Whitney U. Comparisons of percentages were performed using Chi-Square test or (Fisher’s exact test, as appropriate). Ordinal and binomial multivariable logistic regression analyses were performed to analyse the effect of health mediation intervention, to take into account age, gender, HMr and ED. All the tests were two-sided, the statistical significance was defined as *p* < 0.05. Statistical analysis was performed using IBM SPSS Statistics version 20 (IBM SPSS Inc., Chicago, IL, USA).

## Results

**Fig. 1:**
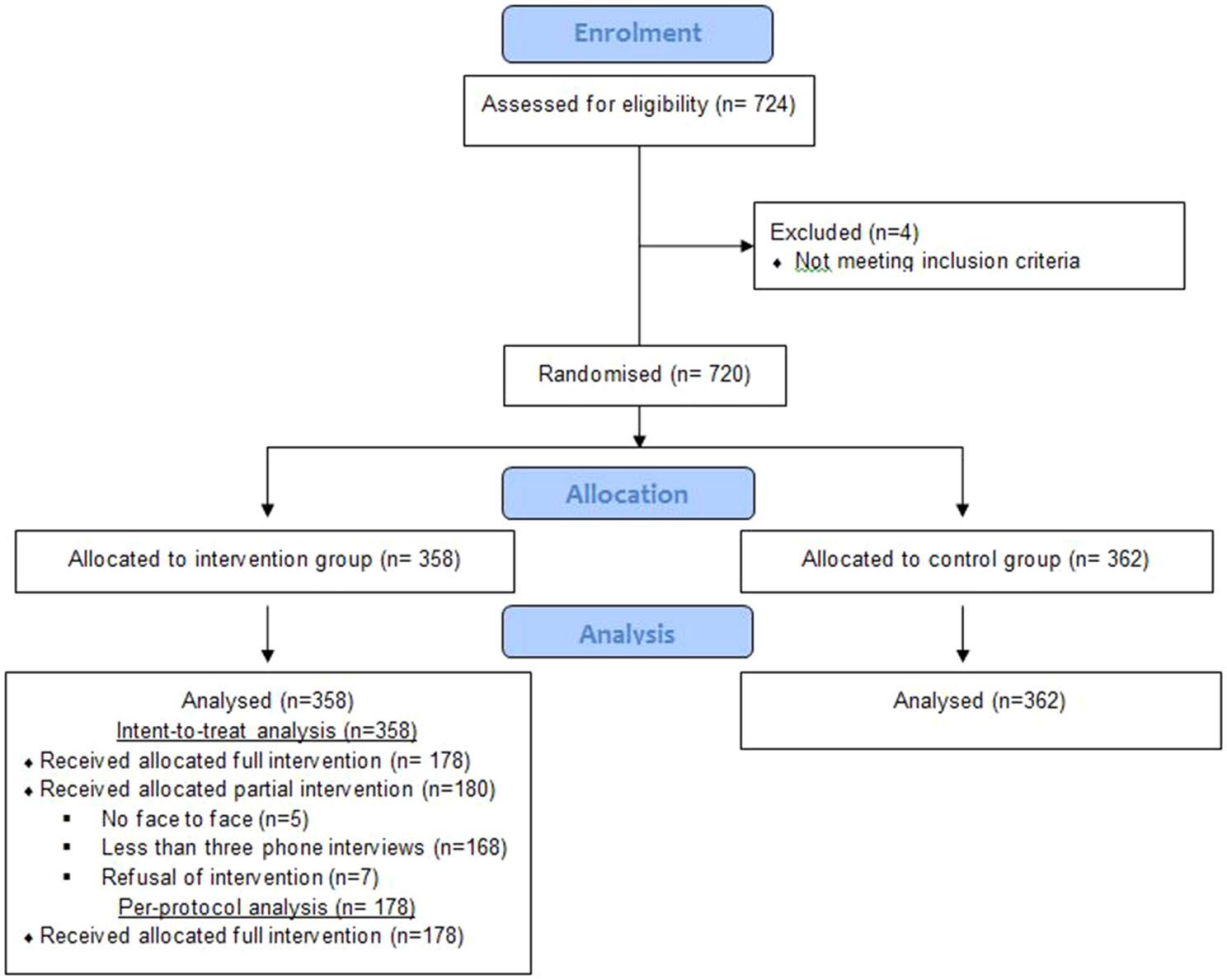
Flow diagram

Among patients assessed for eligibility, 720 patients were included; 178/358 (49.7%) patients of the intervention group were included in the per-protocol analysis (Fig. 1). With regard to precariousness, it is noteworthy that 217 patients (30.6%) had no qualifications, 95 (13.4%) were illiterate, 236 (33%) had not always lived in France, 243 (33.9%) lived alone, 270 (53.3%) were unemployed and 409 (56.8%) were in a very precarious situation (Table 2). With regard to healthcare, 320 (49.3%) had full health insurance coverage for a chronic illness and 86 (12%) received a disability allowance, 96 (13.4%) did not have a GP, 529 (74.8%) had consulted their GP in the previous three months, 375 (71.4%) had reported having no difficulty in contacting their GP (Table 3). All domains of quality of life estimated by the WHO-QOL-BREF were below the average measured in a representative sample of the French population [41]: physical health (51.7 vs.76.9), psychological health (61.3 vs. 67), social relationships (67.6 vs. 74.5) (Table 4).

**Table 2:** Socio-demographic characteristics in the intent-to-treat and per-protocol groups (at enrolment)

	Intent-to-treat (n = 720)		Per-Protocol (n = 540)	
	n	%	n	%
Women	387	53.8	295	54.6
Age	n	%		
* First quartile (18–29 y.)*	183	25.5	129	23.9
* Second quartile (30–47 y.)*	175	24.3	137	25.4
* Third quartile (48–63 y.)*	180	25.0	138	25.6
* Fourth quartile (64–96 y.)*	181	25.2	135	25.0
Education				
* General training - Baccalaureate (A-level)*	267	37.6	199	37.4
* Professional training*	226	31.8	169	31.8
* Without any degree*	217	30.6	164	30.8
Illiterate	95	13.4	75	14.1
Resident in France				
$\le$*5 y.*	37	5.2	29	5.4
* > 5y.*	199	27.7	157	29.1
* Always*	482	67.1	353	65.5
Living				
* Alone*	243	33.9	185	34.5
* With family or friends*	207	28.9	199	28.5
* With a partner*	266	37.2	153	37.1
Occupation (time)				
* Full-time Occupation*	156	30.8	115	30.0
* Part-time occupation*	59	11.6	47	12.3
* No occupation*	270	53.3	221	57.7
Occupation (type*)*				
* Agriculture*	74	10.4	48	9.0
* Employee*	360	50.7	283	53.1
* Blue-collar worker*	106	14.9	79	14.8
* Retired or unemployed*	170	23.9	123	23.1
Level of precariousness				
* Precarious (EPICES Score 30–59)*	409	56.8	303	56.1
* Very precarious (EPICES Score 60+)*	311	43.2	237	43.9

**Table 3:** Access to health care prior to first ED admission and quality of life, in the intent-to-treat and per-protocol groups (at enrolment)

	Intent-to-treat (n = 720)		Per-Protocol (n = 540)	
	n	%*	n	%*
Receives disability allowance	86	12.0	70	13.0
Has a complete insurance coverage for chronic illnesses	320	49.3	240	49.7
Difficulty to reach the attending physician				
* Very easy*	326	45.7	250	46.6
* Easy*	157	22.0	118	22.0
* Not easy*	135	18.9	96	17.9
* No attending physician*	96	13.4	72	13.4
Time since the last visit to the attending physician < 3months	529	74.8	401	75.7
Difficulty to ask questions to the attending physician				
* Always or sometimes difficult*	104	19.8	75	19.0
* Never difficult*	375	71.4	288	72.9
* Don’t know or no answer*	46	8.8	32	8.1
	Mean (std)		Mean (std)	
WHO-QOL domains				
* Physical Health*	51.7(16.7)		52.0(17.2)	
* Psychological Health*	61.3(20.4)		61.6(20.4)	
* Social Relations*	67.6(28.4)		67.8(29.2)	

*(%) among respondents only

Half of the patients lived less than 15 min from the ED, 303 (42.2%) had travelled by ambulance and 120 (16.7%) had walked or used public transportation (Table 4). The main reason given by 326 (45.7%) patients for attending the ED was that they felt their life was in danger, followed by the fact that they had been referred to the ED by the emergency medical dispatch centre or their GP (301: 42.1%). On arrival in ED, 301 patients (40%) were considered by the medical staff to be in a serious condition (injury status and/or functional prognosis deemed likely to worsen in the ED, but not life-threatening, or life-threatening pathological situation with or without immediate resuscitation) (Table 4). Cardiovascular events (188: 26.1%) and traumatic/rheumatologic disorders (152: 21.1%) were the most frequent causes (Table 4). 142 patients (19.8%) were hospitalised following their first admission to the ED (at enrolment). The proportion of patients with low severity score (CCMU 1) at admission in our sample (46 patients: 6.4%) was lower than in the regional average (9.4%). The per-protocol and intent-to-treat populations did not differ in any of the characteristics measured.

**Table 4:** Admission to emergency departments (ED) in the intent-to-treat and per-protocol groups (at enrolment)

	Intent-to-treat (n = 720)		Per-Protocol (n = 540)	
	n	%	n	%
Admitted to ED during night hours	161	22.4	113	20.9
Distance between ED and housing				
* < 15 min*	334	48.8	251	48.7
* 15–30 min*	249	36.4	183	35.5
* > 30 min*	101	14.8	81	15.7
Means of transportation to ED				
* By foot or public transport*	120	16.7	86	16.0
* Personal vehicle*	295	41.0	225	41.8
* Ambulance*	303	42.2	227	42.2
Arrived to ED alone	443	62.0	331	61.6
Reason for coming to ED				
* Called the medical emergency dispatch centre and was told to come to the ED (or were sent a vehicle to be picked up).*	225	31.5	168	31.5
* Don’t have a GP*	12	1.7	8	1.5
* GP told you to go to the ED*	76	10.6	57	10.7
* GP was not available or was unreachable*	20	2.8	17	3.2
* Thought your health was in danger*	326	45.7	240	44.9
* Needed a test that you couldn’t get quickly*	24	3.4	21	3.9
* Lived near the ED*	8	1.1	6	1.1
* Other reason*	29	4.9	23	4.1
ED severity score at ED admission				
* 1- Clinical condition considered stable. No additional diagnostic or therapeutic procedures. Simple clinical examination.*	45	6.4	37	7.0
* 2- Stable lesion status and/or functional prognosis. Decision to perform additional diagnostic or therapeutic procedures in an ED*	358	50.9	259	49.0
* 3- Injury status and/or functional prognosis deemed likely to worsen in the ED, but not life-threatening.*	266	37.8	203	38.4
* 4–5 - Life-threatening pathological situation with or without immediate resuscitation.*	35	5.0	30	5.7
Organic apparatus concerned				
* Gastro-intestinal and Genito-Urinary*	131	18.2	85	15.7
* Cardiovascular*	188	26.1	139	25.7
* Respiratory*	82	11.8	67	12.4
* Trauma and rheumatology*	152	21.1	117	21.7
* Psychiatric/Intoxication*	68	9.4	51	9.4
Hospitalised after discharge from the ED	142	19.8	115	21.3

The proportion of patients who were readmitted at 90 days was high but did not differ between the control and the HM intervention groups (31.7% vs. 36.3, *p* = 0.23) (Table 5). There was no significant difference between the control and HM intervention groups for any of the secondary outcome measures (Table 5). The per-protocol analysis also did not identify any significant difference for the primary and secondary endpoints (Table 5). The same analyses, performed only in patients with a low severity score at enrolment, and then in those with a high severity score (EPICES score 60+) showed no significant differences between the control and HM intervention groups for any of the outcomes measured.

**Table 5:** Readmissions and re-hospitalisations to ED according to the health mediation intervention

		Intent-to-treat		Per-Protocol	
	Control group	Health Mediation intervention	p	Health Mediation intervention	p
n	362	358		178	
**Admitted to ED at 30 days**			0.24		0.29
* No*	295 (81.5%)	279 (77.9%)		136 (76.4%)	
* Once*	47 (13%)	49 (13.7%)		25 (14.0%)	
* Twice or more*	20 (5.5%)	30 (8.4%)		17 (9.6%)	
Admitted to ED at 30 days for the same reason	25 (6.9%)	38 (10.6%)		19 (10.7%)	
Number of readmissions at 30 days	0.30 (0.94)	0.37 (0.88)	0.32	0.39 (0.85)	0.32
**Admitted to ED at 90 days**					
*No*	247 (68.2)	228 (63.7%)	0.23	111 (62.4%)	0.33
*Once*	66 (18.2)	81 (22.6%)		38 (21.3%)	
*Twice or more*	49 (13.5)	49 (13.7%)		29 (16.3%)	
Admitted to ED at 90 days for the same reason	25 (6.9)	23 (6.4%)	0.79	11 (6.2%)	0.80
ED severity score at ED admission at 90 days			0.99		0.37
* 1- Clinical condition considered stable. No additional diagnostic or therapeutic procedures. Simple clinical examination* ***Or*** *2- Stable lesion status and/or functional prognosis. Decision to perform additional diagnostic or therapeutic procedures in an ED*	47 (65.3%)	50 (65.8%)		26 (61.9%)	
* 3- Injury status and/or functional prognosis deemed likely to worsen in the ED, but not life-threatening.*	23 (31.9%)	23 (30.3%)		14 (33.3%)	
* 4–5 - Life-threatening pathological situation with or without immediate resuscitation.*	2 (2.8%)	3 (3.9%		2 (4.8%)	
Number of readmissions at 90 d.	0.67 (1.6)	0.71 (1.5)	0.69	0.80 (1.6)	0.37
Number of hospitalisations at 90 d.	15 (4.1)	16 (4.5)	0.83	11 (6.2%)	0.29
**Admitted to ED at 180 days**			0.18		0.25
* No*	207 (57.2%)	194 (54.2%)		95 (53.4)	
* Once*	80 (22.1%)	83 (23.2%)		38 (21.3)	
* Twice or more*	75 (20.7%)	81 (22.6%)		45 (25.3)	
Admitted to ED at 180 days for the same reason	7 (1.9%)	14 (3.9%)	0.12	8 (4.5)	0.09

P-adjusted for age, gender, emergency department and health mediator

## Discussion

While our previous social-psychological analysis showed that both FUED living in precarious conditions and ED professionals recognised the needs to address bio-psycho-social distress and the utility of HM [36], the results of our randomised trial showed no effect of HM on 90-day readmission rates or any of the secondary outcomes.

We compared the characteristics of the patients enrolled in our randomised trial with the available data from the EDs of the same region [37], and when not available to the national data, with the caveat that the enrolment period included the Covid pandemic and the lockdown periods. First, the proportion of women and the mean age were higher in our sample than those reported by the EDs in the same region, 53.8% vs. 48.4%, 47.8 vs. 45 years respectively [37]. The proportion of patients with a low admission severity score (CCMU 1) in our sample was lower than the regional average (6.4% vs. 9.4%). In contrast, the proportion of patients with a high severity score (CCMU 4–5) was higher in our sample than in the region (5% vs. 2.0%) while ours were less likely to be admitted to hospital at ED discharge (19.8% vs. 24.9%). We also found that the proportion of patients admitted to the EDs for trauma was lower in our sample than in the region (11.5% vs. 29%), while the proportion of patients admitted for psychiatric or toxicological reasons was higher (9.4% vs. 4%). All domains of quality of life estimated by the WHO-QOL-BREF were below the average in a representative sample of the French population [41]. Relative to regional and national observations, the FUED in our sample were more likely to be women, older, have psychiatric disorders and a poorer quality of life, more likely to be admitted for serious health and vital conditions, and less likely to be admitted for trauma, which is consistent with the literature data [5–10]. It is interesting to note that most FUED in our sample had a GP and had consulted a GP in the last three months, indicating a precarious state of health rather than difficulties in accessing healthcare.

Two main methodological issues should be discussed to explain the lack of effect of HM on the readmission rates at 30, 90 and 180 days, the first concerning the characteristics of the patients enrolled in the trial and the second concerning the HM intervention.

### Patient profile may help explain why HM did not work

A recurring methodological issue in the evaluation of interventions is how to define a FUED [13]. The definition generally varies between 3 and 5 admissions per year [9, 42–44]. Our study used a rather low criterion (at least 3 ED admissions per year). However, 44.3% of the patients enrolled in our trial were readmitted at least once at 180 days (including 21.6% at least twice). Another issue is the heterogeneity of patients and their reasons for presenting to the ED raised by Raven et al. [13]. In our sample, the reasons for ED re-use were very diverse: 25.6% had returned to the ED for cardiovascular reasons, while 21.7% had come for trauma or rheumatological problems. It should also be noted that 42% of the patients had been referred by an emergency medical regulation department or a general practitioner, and 44.9% had felt in danger on first presentation. It is uncertain whether an HM intervention can be effective in these patients. Rather than using this criterion alone (being a FUED and living in precarious conditions), interventions should start with a thorough social, psychosocial and health assessment of whether and how an intervention is likely to prevent repeat admissions, as a result of an accurate joint medical and social assessment, and therefore to target only these FUED.

Another issue is the definition of precariousness and its use to attribute an intervention to a FUED. Several terms or dimensions have often been used in papers investigating the factors related to iterative ED use: social vulnerability, deprivation, unemployment, economic hardship…. The causes and dimensions of precariousness are multiple and affect heterogeneous populations [45]. Precariousness appears to be a multidimensional dynamic process, the result of a series of events experienced in different areas of an individual’s life (housing, employment, culture, education, family and social relations, physical and mental health…) [46, 47]. In our trial, we chose the EPICES score which has demonstrated its value in identifying people in precarious situations who are at increased risk of health problems and are not recognised by the criteria of the socio-administrative definition [48]. Based on this score, we enrolled patients with varied vulnerabilities (social and material deprivation, health and financial difficulties), some of which can be reached only by a long-term HM, and/or accepted by patients. Even if the diagnosis of needs made by the HMs allowed the intervention to be tailored, it is likely that some patients did not feel sufficiently concerned or motivated by an intervention offered during their visit to the ED. Half of the patients in the intervention arm did not accept more than one face-to-face meeting, had fewer than three telephone contacts and/or did not follow up with the HM, which highlights the difficulty of engaging a majority of patients in HM. HM should be offered only to patients who are able or fully willing to benefit from it. Therefore, the concept of precariousness does not seem to be the only criterion to be used in the evaluation of interventions aimed at reducing the use of emergency services. A detailed assessment of the components of precariousness would be more appropriate for selecting FUED who could be helped by an intervention aimed at reducing the risk of repeated use of ED.

### Is health mediation an appropriate tool to reduce the iterative use of EDs?

Initially, HM focused on persons living with AIDS and mental health diseases, and then expanded to patients with other chronic conditions, but was not designed for FUEDs [16, 23]. In our trial, 13.4% reported not having a GP, a slightly higher proportion than in the general population, and 24.3% had not seen a GP in the last three months, despite a higher prevalence of chronic diseases and a poorer quality of life than the general population. These findings mean that less than a quarter of our sample needed strong support. In future evaluations, HMs should focus their attention and resources on the most vulnerable patients, who are the hardest to reach. In the qualitative analysis of the intervention, we noticed that many deprived persons discontinued their telephone subscription because of financial issues, had technical difficulties to listen to their voice messages, and faced constraints/barriers to respect their appointments [36]. The COVID 19 pandemic and its lockdown periods also affected the organisation and the burden of EDs and HM, and enhanced the isolation of patients and the difficulties of remaining in contact with them. The HMrs were obliged to favour phone contacts instead of face-to-face meetings, and several patients were lost to follow up. Our experience highlights the need for a very high level of investment in human resources and the need to identify and focus on the most vulnerable patients, which also raises the issue of stigma and the need for clear explanations to patients when HM is offered [44].

Another difficulty we encountered was working with the medical and social staff of the ED. The HMrs were present in the ED, in contact with the ED staff and very well accepted [36]. However, the HMrs did not always manage to involve the ED medical and social staff sufficiently in the discharge care plan, mainly because of the work overload. The ED staff showed high expectations of HMrs, but several ED professionals reported that they were faced with intense assignments and a high psycho-emotional load on a daily basis, which led to feelings of dissatisfaction when caring for the FUED living in precarious conditions. A great deal of training and support is needed for ED staff, and it is essential that HMrs are fully integrated into the ED staff so that decisions about treatment take into account precariousness and social vulnerability.

In their scoping review, Richard et al. identified the conditions for the success and the feasibility of HM, in particular the status and training of HMrs [23]. They also noted that most papers took the effectiveness of HM for granted and only presented an analysis of the conditions. It is now essential to define the profile of patients who can benefit from HM in order to improve the health care indicators, including ED use, as well as the methods and duration of HM. Our intervention was designed for a maximum support period of 90 days to achieve our main objective of reducing ED readmission. Our qualitative analysis showed that some patients would have liked a longer period of support, which would have allowed them to be better managed in the long term in outpatient settings [36]. The lack of evidence that HM reduces the number of ED re-use highlights the difficulty to involve patients living in precarious conditions. A longer-term intervention based on a more holistic approach and targeting both the capacities of individuals and the environmental conditions in which they find themselves may be more effective in reducing the frequent use of emergency departments in the long term [49, 50].

## Conclusion

Although health mediation seemed to be a promising solution at the end of our qualitative study [36], our study did not show that it was effective in reducing the use of emergency services by vulnerable frequent users of ED. Interventions targeting this population living in precarious conditions should aim to accurately identify their social, psychosocial and medical needs, involve ED staff and train them on the issue of precariousness, with a view to long-term health empowerment.

## Data Availability

The data that support the findings of this study are not openly available due to reasons of sensitivity and are available from the corresponding author upon reasonable request.

## Abbreviations

AIDS: Acquired Immune Deficiency Syndrome
CHW: Community Health Worker
CM: Case Management
COVID: COronaVIrus Disease
ED: Emergency Department
EPICES: Evaluation de la Précarité et des Inégalités de santé dans les Centres d’Examens de Santé (Evaluation of precariousness and health inequalities in health examination centers)
HM: Health Mediation
HMrs: Health Mediators
WHOQOL-BREF: World Health Organization Quality Of Life
