# Correction: Health mediation does not reduce the readmission rate of frequent users of emergency departments living in precarious conditions: what lessons can be learned from this randomised controlled trial?

**DOI:** 10.1186/s12873-024-01110-x

**Published:** 2024-10-12

**Authors:** Michel Rotily, Nicolas Persico, Aurore Lamouroux, Ana Cristina Rojas-Vergara, Anderson Loundou, Mohamed Boucekine, Themistoklis Apostolidis, Sophie Odena, Celia Chischportich, Pascal Auquier

**Affiliations:** 1 Centre d’Etudes et de Recherche sur les services de santé et la qualité de vie (CEReSS), Aix Marseille Université, Marseille, France; 2 Service des Urgences, Hôpital Nord, Assistance Publique Hôpitaux de Marseille, Marseille, France; 3 Centre de santé hospitalo-universitaire des Aygalades, Assistance Publique Hôpitaux de Marseille, Marseille, France; 4 Laboratoire de Psychologie Sociale (LPS), Aix Marseille Université, Aix en Provence, Marseille, France; 5 Aix Marseille Univ, CNRS, LEST, Aix-en-Provence, France; 6 SCOP Regards Santé, Marseille, France

**BMC Emergency Medicine (2024) 24:83**

10.1186/s12873-024-01000-2

Affiliation #5 in the original article is erroneously presented; the affiliation information should instead state the following:

Aix Marseille Univ, CNRS, LEST, Aix-en-Provence, France

